# Danger-Pose Detection System Using Commodity Wi-Fi for Bathroom Monitoring

**DOI:** 10.3390/s19040884

**Published:** 2019-02-20

**Authors:** Zizheng Zhang, Shigemi Ishida, Shigeaki Tagashira, Akira Fukuda

**Affiliations:** 1 Graduate School/Faculty of Information Science and Electrical Engineering, Kyushu University, Fukuoka 819-0395, Japan; ishida@f.ait.kyushu-u.ac.jp (S.I.); fukuda@f.ait.kyushu-u.ac.jp (A.F.); 2 Faculty of Informatics, Kansai University, Osaka 569-1095, Japan; shige@res.kutc.kansai-u.ac.jp

**Keywords:** channel state information (CSI), non-line-of-sight (NLOS), one-class support vector machine (SVM), anomaly detection, healthcare

## Abstract

A bathroom has higher probability of accidents than other rooms due to a slippery floor and temperature change. Because of high privacy and humidity, we face difficulties in monitoring inside a bathroom using traditional healthcare methods based on cameras and wearable sensors. In this paper, we present a danger-pose detection system using commodity Wi-Fi devices, which can be applied to bathroom monitoring, preserving privacy. A machine learning-based detection method usually requires data collected in target situations, which is difficult in detection-of-danger situations. We therefore employ a machine learning-based anomaly-detection method that requires a small amount of data in anomaly conditions, minimizing the required training data collected in dangerous conditions. We first derive the amplitude and phase shift from Wi-Fi channel state information (CSI) to extract low-frequency components that are related to human activities. We then separately extract static and dynamic features from the CSI changes in time. Finally, the static and dynamic features are fed into a one-class support vector machine (SVM), which is used as an anomaly-detection method, to classify whether a user is not in bathtub, bathing safely, or in dangerous conditions. We conducted experimental evaluations and demonstrated that our danger-pose detection system achieved a high detection performance in a non-line-of-sight (NLOS) scenario.

## 1. Introduction

For people living alone, especially elderly and disabled people, health monitoring has become one of the important topics in daily life. Health-monitoring methods, such as fall detection and heart rate monitoring, are performed to monitor users’ health condition. According to surveys from the Japanese Society of Legal Medicine and The Japanese Society of Balneology, Climatology and Physical Medicine, 20,000 people on average die in bathrooms every year in Japan [1,2]. In particular, 70% of the deaths were drowning deaths, which were mainly caused by losing consciousness or by heart disease, when people fell down or the body temperature changed suddenly, respectively. If we can detect a danger pose that will cause drowning and perform a treatment such as bathtub flush, fatal accidents might be decreased.

Our goal is to detect dangerous conditions in a bathroom and to perform some actions to reduce fatal accidents in a bathroom. Although there are many health-monitoring systems in use, bathroom monitoring is unavailable for now as the bathroom is highly private space. Health monitoring using traditional methods such as computer vision or wearable devices are relatively mature [3,4]. However, due to the high privacy of the bathroom, computer vision methods might invade users’ privacy and might also be affected by fog. Wearable devices are not suitable for health monitoring in a bathroom because of the lack of comfort.

As a first step toward our goal, in this paper, we present a real-time danger-pose detection system using Wi-Fi signals to detect dangerous poses in a bathtub caused by asphyxia or unconsciousness. Our danger-pose detection system uses Wi-Fi radio signals for monitoring in a bathroom while preserving privacy. Wi-Fi orthogonal frequency division multiplexing (OFDM) communication [5] with a multiple input multiple output (MIMO) technique [6] calculates radio channel responses on many subcarriers for communication, which can be derived as channel state information (CSI) on IEEE 802.11n/ac compliant devices. We use the CSI to monitor changes in radio channel response detecting danger poses.

Wi-Fi CSI-based detection methods have been successfully applied to many applications, such as fall detection and respiration detection [7,8]. These methods fully rely on machine learning algorithms that require training data for each detection target situation. Our detection target is dangerous conditions, where data collection is difficult.

We use an anomaly-detection algorithm that requires no data in dangerous conditions. We first derive amplitude and phase shift from the CSI and extracted low-frequency components that are related to human activities. We then extract the static and dynamic features, which are fed into a one-class support vector machine (SVM)-based classifier, an anomaly-detection algorithm, to detect danger situations of a user in a bathroom. We conducted experimental evaluations in a bathroom in an apartment with many residents on six separate days over a three-month period and revealed that the danger-pose detection system successfully detected dangerous situations with a recall of 96.23%.

The main contributions of this paper are summarized below.

- We verify the feasibility of Wi-Fi CSI sensing for danger detection in a bathroom environment, especially in a bathtub. To the best of our knowledge, this is a first work to use CSI for anomaly detection.
- We propose a dynamic feature extraction method of CSI, which effectively reduces the influence of environmental changes.
- We only rely on training data derived in normal situations. We collect data when a user is normally bathing/showering and feed them into an anomaly-detection algorithm to avoid collecting the data in dangerous situations, which is difficult to collect in practical use.
- We measured the performance of this method in an actual environment. The experimental results show that the danger-pose detection system has high detection performance in a non-line-of-sight (NLOS) scenario.

The remainder of this paper is organized as follows. We introduce related work in Section 2. In Section 3, we design the danger-pose detection system that can be applied to bathroom monitoring. We conducted experiments to evaluate our system in Section 4. Section 5 concludes the paper and envisages the future work.

## 2. Related Work

To the best of knowledge, this is a first attempt at anomaly detection using Wi-Fi CSI. In this section, we briefly look through related work on healthcare and wireless sensing.

### 2.1. Traditional Healthcare Methods

Traditional healthcare methods are categorized into three types: computer vision-based, wearable device-based, and ambient sensor-based methods [9,10].

The computer vision-based methods use low cost RGB cameras and depth cameras to estimate the state of a user [11]. Miguel et al. presented an RGB camera-based fall-detection system, which is a device-free method [12]. The fall-detection system achieved high detection performance with a detection rate of more than 96%. Lotfi et al. proposed a vision-based fall-detection method that deals with occlusion problem by using head tracking [10]. Balakrishnan et al. proposed a camera-based heart rate monitoring method using subtle head motions caused by a pulse [13]. Experimental evaluation demonstrated that the detected heart rate was almost the same as the heart rate measured by an electrocardiogram device. The computer vision-based methods invade users’ privacy, which is critical for monitoring in a bathroom. The computer vision-based methods also receive environmental constraints: there will be blind spots in an NLOS environment.

The wearable device method uses wearable sensors such as accelerometer, gyroscope, light intensity sensor to detect users’ safety state while preserving users’ privacy. Jain et al. presented a portable fall-detection device attached to a neck of a vest [14]. Sabatini et al. presented a fall-detection method using inertial sensor and barometer [15]. These methods calculate root-mean-square accelerometer data and/or other features and detect the big impact. An obvious shortcoming of the wearable device methods is a deterioration in comfort of users when taking a bath. Wearable devices could also be forgotten to wear, especially by dementia patients.

The ambient sensor-based methods use sensors such as sound, vibration, pressure, and radar embedded in an environment to recognize human activities. Zigel et al. presented a fall-detection system relying on sound and vibration generated by a fall event [16]. However, the ambient sensor-based methods suffer from high false detections due to noise such as everyday object falls [17].

Radar-based device-free methods have also been proposed [18,19]. The radar-based methods require a line-of-sight (LOS) environment, i.e., no occlusion is allowed between radar and a user, which highly restricts physical deployment.

### 2.2. Sensing Using Wireless Communication Signals

In recent years, the use of wireless signals for sensing has shown great potential in the field of device-free sensing. Major strength of wireless sensing is that the wireless sensing can be applied not only to LOS environments but also to NLOS environments. Wireless sensing technologies are well studied for indoor localization and motion recognition.

As an example of traditional wireless sensing technologies, Sugano et al. presented an indoor localization system using received signal strength (RSS) in a wireless sensor network [20]. A recent paper presented Chronos, which is an indoor localization system that achieves decimeter-level localization using a single Wi-Fi access point [21]. Chronos calculates distances between multiple antennas and a target device based on time of flight of Wi-Fi signals using channel state information (CSI) derived from commodity Wi-Fi devices. Chronos is comparable to state-of-the-art localization systems that require four or five access points.

CSI-based sensing methods have also been proposed. Ali et al. designed a keystrokes recognition method using CSI [22]. Keystroke features are extracted by performing principal component analysis (PCA) on CSI to locate fingers pushing keys. The keystroke recognition method achieves a word recognition accuracy of more than 85%. RT-Fall is a fall down detection system using Wi-Fi signals [7]. RT-Fall uses phase difference between antennas for fall detection and achieves high accuracy. RF-Pose is a through-wall human pose estimation method using radio signals [23]. RF-Pose draws heatmaps in two dimensions by a deep neural network to estimate human posture beyond walls. Experimental evaluations reveal that the RF-Pose can describe human posture in detail.

However, detection in bathroom, especially in a bathtub, is still challenging:

- Bath water might cover human body and let body become unrecognizable by wireless signals.
- Training data in dangerous situations for a machine learning algorithm is hard to obtain. We can simulate danger poses and get data for evaluation, which are not suitable as training data in practical use.
- Due to the lack of training data, over fitting is a problem that should be solved for bathroom monitoring.

## 3. Danger-Pose Detection System in Bathroom

### 3.1. Overview

The danger-pose detection system uses a two-step estimation process. In the first step, we detect whether a user is taking a bath based on static features. In the second step, we estimate whether the user is in safe or in danger based on dynamic features only when the user is detected as “taking a bath” in the first step.

**Figure 1:**
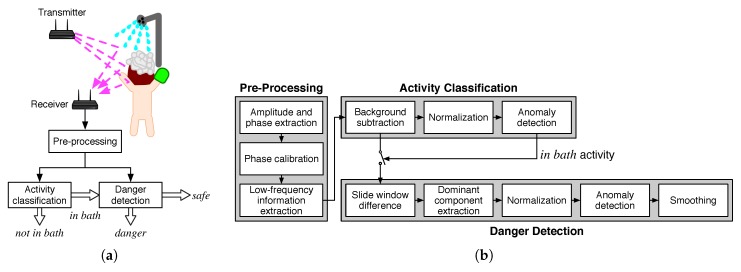
System architecture: (**a**) outline of system architecture, and (**b**) system components.

Figure 1 shows a system architecture of our danger pose detection system. As shown in Figure 1a, the danger-pose detection system consists of pre-processing, activity classification, and danger detection blocks as well as transmitter and receiver. We install transmitter and receiver in an NLOS environment, i.e., outside of a bathroom. Wi-Fi signals sent from the transmitter are reflected and diffracted by a monitoring target and reach the receiver. We extract CSI from the receiver, which are passed to the pre-processing block where calibration and denoising processes are performed to extract information related to human activities.

The activity classification block performs the first step estimation, which extracts static features for coarse estimation of user activity. In this step, we only detect whether a user is in a bathtub, which is referred as *in bath* activity in this paper.

In case that the user is *in bath* activity, the danger detection block performs the second step estimation, which estimates user situations within *safe* or *danger*. The *danger* situation represents that the user is in a coma or in situations where the user cannot perform any activity, while the *safe* situation represents not in danger situations.

In the following subsections, we describe the each block in more detail.

### 3.2. Pre-Processing

As shown in Figure 1b, the pre-processing block consists of amplitude/phase extraction, phase calibration, and low-frequency information extraction processes.

#### 3.2.1. Amplitude and Phase Extraction

In the amplitude and phase extraction process, amplitude and phase shift information is extracted from raw CSI data. In an indoor environment, because there are many objects including walls and ceiling, wireless signals reach a receiver after multiple reflections and diffractions, which we call multipath. Multipath causes amplitude attenuation and phase shift, resulting in distortion of the wireless signals. The receiver therefore calculates and compensates for the distortion using known signals named pilot symbols embedded in each transmission. The distortion information can be extracted as CSI in recent IEEE 802.11n/ac compliant devices.

The CSI is actually a radio channel frequency response of each subcarrier in OFDM communication. Subcarriers are essentially a series of carrier waves with slightly different frequencies. The channel frequency response of the *s*-th subcarrier can be written as:

(1) $$H_{s}=A_{s}e^{j\phi_{s}},\;s\in \left[1,30\right],$$

where $A_{s}$ and $\phi_{s}$ denote the amplitude and phase shift of *s*-th subcarrier, respectively. The CSI measurement at time *t* can be expressed as:

(2) $$H\left(t\right)=[H_{1}\left(t\right),H_{2}\left(t\right),\cdots ,H_{30}\left(t\right)].$$

MIMO technology uses multiple antennas for data transmission and reception, which improves data throughput without increasing bandwidth and transmission power. The MIMO-OFDM composite technology is one of the adopted standards of IEEE 802.11n. Assume that there are *n* transmission antennas and *m* reception antennas. CSI for the wireless signals sent on different subcarriers from different antennas can be expressed as:

(3) $$\mathrm{CSI}\left(t\right)=\left[\begin{array}{cccccc} H_{1,1,1}\left(t\right) & \cdots & H_{1,1,30}\left(t\right) & H_{1,2,1}\left(t\right) & \cdots & H_{1,m,30}\left(t\right)\\ H_{2,1,1}\left(t\right) & \cdots & H_{2,1,30}\left(t\right) & H_{2,2,1}\left(t\right) & \cdots & H_{2,m,30}\left(t\right)\\ \vdots & \ddots & \vdots & \vdots & \ddots & \vdots \\ H_{n,1,1}\left(t\right) & \cdots & H_{n,1,30}\left(t\right) & H_{n,2,1}\left(t\right) & \cdots & H_{n,m,30}\left(t\right) \end{array}\right],$$

where $H_{n,m,s}\left(t\right)$ is the CSI of the *s*-th subcarrier between *n*-th transmission and *m*-th reception antennas.

As for CSI extraction, we use the Linux 802.11n CSI Tool [24]. The CSI signal extracted by CSI Tool is stored in complex form. We can extract the amplitude and phase information on the original CSI as shown in Equation (1). Although IEEE 802.11n uses at least 64 subcarriers, we can derive CSI only for 30 subcarriers, which is a limitation of the CSI Tool we used.

#### 3.2.2. Phase Calibration

The phase information cannot be used directly due to the random noise caused by hardware imperfection and uncoordinated delay between transmitter and receiver [8,25]. The phase information contains interference from environmental noise and hardware defects as:

(4) $$\hat{\phi_{s}}=\phi_{s}+2\pi f_{f}\Delta t+\beta +Z,$$

where $\hat{\phi_{s}}$ is the CSI phase measurement of subcarrier *s*, $\phi_{s}$ is the genuine phase, $f_{f}$ is the carrier frequency offset at the receiver, and Δt is the time lag due to sampling frequency offset. β is unknown phase offset caused by carrier frequency offset and *Z* is measurement noise.

We perform phase calibration algorithm presented in [26]. For convenience, we define the slope ξ of phase and the offset *b* across the entire frequency band defined as:

(5a) $$\begin{array}{cc} \xi & =\frac{\hat{\phi_{30}}-\hat{\phi_{1}}}{m_{30}-m_{1}}, \end{array}$$

(5b) $$\begin{array}{cc} b & =\frac{1}{30}\sum_{s=1}^{30}\hat{\phi_{s}}, \end{array}$$

where $m_{s}$ is the subcarrier index of subcarrier *s* defined in IEEE 802.11 standard [27]. The calibrated phase $\tilde{\phi_{s}}$ can be calculated by:

(6) $$\tilde{\phi_{s}}=\hat{\phi_{s}}-\xi m_{s}-b.$$

**Figure 2:**
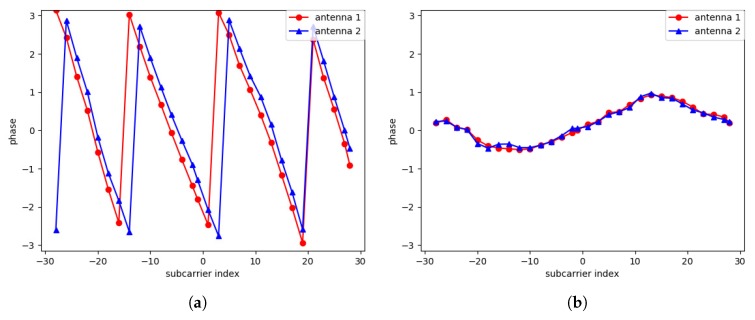
The result of phase calibration: (**a**) raw phase measurement, and (**b**) calibrated phase.

Figure 2 shows the effect of the calibration.

#### 3.2.3. Low-Frequency Information Extraction

According to the sampling theorem, a series of time-varying CSI contains signals of frequency components from 0 to $\left(f_{s}/2\right)$ Hz, where $f_{s}$ is a sampling frequency.

We extract low-frequency information to filter out high frequency unnecessary signals. For human motion detection, many redundancies are generated because sampling frequency $f_{s}$ is much larger than the frequency of human activities and there is high frequency noise.

First, we use fast Fourier transform (FFT) to analyze frequency components of CSI:

(7) $$a_{n}=\sum_{k=0}^{N-1}A_{k}e^{j\frac{2\pi }{N}kn},\;n=0,\ldots ,N-1,$$

where $a_{n}$ is the frequency domain CSI amplitude, *N* is the number of samples in FFT, and $A_{k}$ is the CSI amplitude. We also calculate the frequency domain CSI phase.

**Figure 3:**
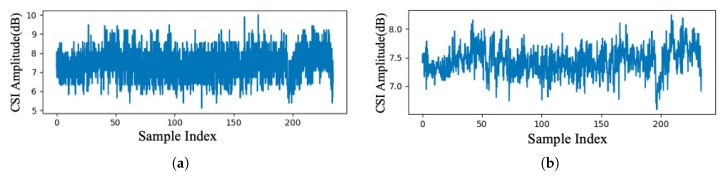
Result of low-pass filtering: (**a**) raw amplitude measurement, and (**a**) amplitude after low-pass filter.

We then extract the low-frequency information from CSI to filter out high frequency noise and information that has small relation with human activities. Based on a preliminary experiment results, we set the cutoff frequency to 2 kHz. We then perform inverse FFT to restore time domain CSI. Figure 3 shows an example of low-pass filtering result.

We describe the CSI derived from pre-processing block as $\tilde{\mathrm{CSI}}\left(t\right)$, which includes the low-pass filtered and calibrated amplitude/phase information.

### 3.3. Activity Classification

In the activity classification block, static features are extracted for coarse activity recognition. As shown in Figure 1b, the activity classification block consists of background subtraction, normalization, and anomaly detection processes.

#### 3.3.1. Background Subtraction

CSI is not only containing human activity information, but also containing environment information that should not be learned in a machine learning activity classifier. As Domenico et al. pointed out in [28], the detection system trained with data derived in a specific environment shows high detection performance in the same environment. However, due to the lack of generalization ability, the environment information as background might cause over fitting, resulting in invalid danger recognition when the environment changes.

**Figure 4:**
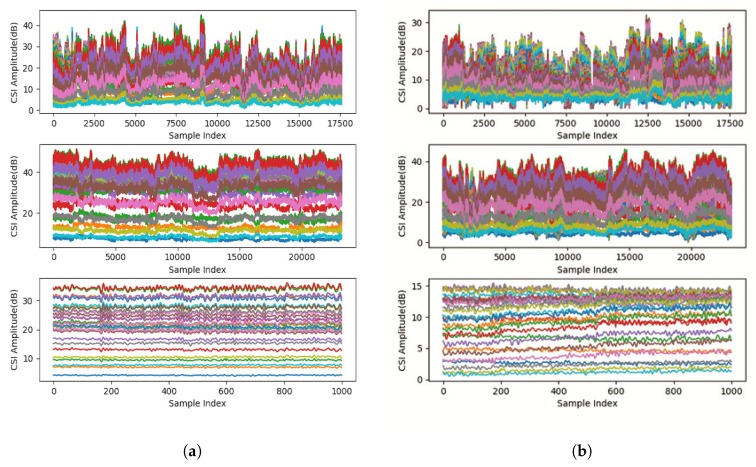
CSI amplitude of 30 subcarriers in shower, safe, and danger situations (from top to bottom). (**a**) CSI amplitude on one reception antenna, and (**b**) amplitude difference between two reception antennas.

We therefore perform background subtraction by extracting difference between reception antennas on each subcarrier. Figure 4 shows CSI amplitude of 30 subcarriers when a user is taking a shower, bathing safely, and bathing dangerously. Please note that the danger pose is simulated. We found that CSI difference of the same subcarrier between different reception antennas is sensitive to human activities but not to environmental changes. We use the CSI difference between reception antennas instead of directly using CSI for background subtraction. Please note that we still should use training data including environmental changes to avoid over fitting to a specific environment.

Here we describe $\tilde{\mathrm{CSI}}\left(t\right)$ using channel frequency response of each reception antenna:

(8) $$\tilde{\mathrm{CSI}}\left(t\right)=\left[{\tilde{H}}_{1,1}\left(t\right),\cdots ,{\tilde{H}}_{1,30}\left(t\right),{\tilde{H}}_{2,1}\left(t\right),\cdots ,{\tilde{H}}_{m,30}\left(t\right)\right],$$

where ${\tilde{H}}_{m,s}={\left[H_{1,m,s},H_{2,m,s},\cdots ,H_{n,m,s}\right]}^{T}$ is the low-pass filtered calibrated channel response of subcarrier *s* on reception antenna *m*. Background subtraction process calculates CSI antenna difference ${\mathrm{CSI}}^{\prime }\left(t\right)$ as:

(9) $${\mathrm{CSI}}^{\prime }\left(t\right)=\left[{\tilde{H}}_{2,1}\left(t\right)-{\tilde{H}}_{1,1}\left(t\right),\cdots ,{\tilde{H}}_{2,30}\left(t\right)-{\tilde{H}}_{1,30}\left(t\right),\cdots ,{\tilde{H}}_{m,30}\left(t\right)-{\tilde{H}}_{m-1,30}\left(t\right)\right],$$

which we call the static features.

#### 3.3.2. Normalization

To improve classification accuracy and reduce the training time, we normalize the CSI data onto a value in a range of [0,1]. CSI amplitude has a lager range of values compared to the phase and will be dominant in the classification process, resulting in degradation of accuracy and training speed. We adopt the MIN-MAX normalization. The normalized value $X_{i}^{*}$ is:

(10) $$X_{i}^{*}=\frac{X_{i}-X_{\min}}{X_{\max}-X_{\min}},$$

where *X* is amplitude *A* or phase ϕ of each element in ${\mathrm{CSI}}^{\prime }\left(t\right)$, $X_{i}$ is the raw data, $X_{\min}$ and $X_{\max}$ are the minimum and maximum values in the original data, respectively. To keep all the information, we choose static values of $X_{\min}$ and $X_{\max}$. Based on a preliminary experiment, we determined the values as $A_{max}=30$, $A_{min}=0$, $\phi_{max}=\pi$, and $\phi_{min}=-\pi$.

#### 3.3.3. Anomaly Detection

The anomaly-detection process finally estimates user activity. Anomaly-detection algorithms are always used in cases that outlier data, i.e., data in dangerous situations in our case, is little or difficult to get. Anomaly detection mainly relies on inlier data in training to detect outliers.

**Figure 5:**
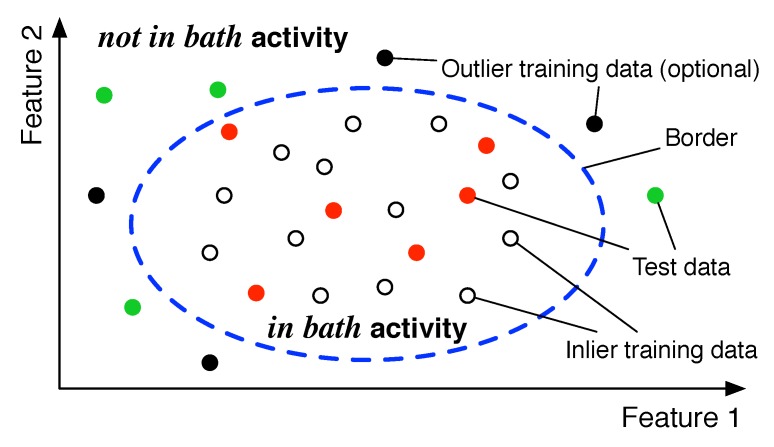
Example of bathroom activity classification using anomaly detection.

Figure 5 shows an example of bathroom activity classification using anomaly detection, suppose that we can distinguish the user activities *in bath* or *not in bath* by two kinds of features. White dots represent inlier training data obtained while a user is taking a bath and showering. Black dots represent outlier training data obtained while a user simulates danger poses in a bathroom, which is optional in anomaly detection. In a training phase, an anomaly-detection algorithm determines a border, indicated by a dashed blue line in the figure, to distinguish *in bath* or *not in bath* using the training data. In a classification phase, we feed test data to the trained anomaly detection algorithm. Red and green dots both indicate test data, which are supposed to be classified into *in bath* and *not in bath* activities, respectively, by the anomaly-detection algorithm.

We do not limit an anomaly-detection algorithm to use in our detection system. In this paper, we use a one-class SVM as an anomaly-detection algorithm. One-class SVM supports one type of training data and maps the data onto a higher domain feature space to put the training data onto the same side of a hyperplane. In a classification phase, the outliers are put onto another side of the hyperplane. Gaussian kernel is used in our one-class SVM classifier to establish a normal distribution model for features of each dimension.

### 3.4. Danger Detection

As shown in Figure 1b, the danger detection block consists of slide window difference, dominant component extraction, normalization, anomaly detection, and smoothing processes. The normalization process is the same process as described in Section 3.3.2.

#### 3.4.1. Slide Window Difference

The classification of *in bath* and *not in bath* is mostly based on location and posture of a user and that is the reason the static features have enough information for activity classification. For danger-pose detection, the location and posture of the user are insufficient.

To detect *safe* or *danger* in a bathtub, we focus on body parts above water because the body parts under water are hard to detect by wireless signals. When a user loses consciousness, the intensity of activities becomes lower than in a normal status. We extract the intensity as *dynamic features* to detect danger poses.

After the background subtraction described in Section 3.3.1, we calculate the CSI difference between two different time to extract dynamic features. We can calculate the CSI difference between time $t_{i}$ and $t_{j}$ as:

(11) $$\mathrm{abs}\left\{{\mathrm{CSI}}^{\prime }\left(t_{j}\right)-{\mathrm{CSI}}^{\prime }\left(t_{i}\right)\right\},\;t_{i}<t_{j},$$

where abs( ) indicates element-wise absolute operation over matrix *X*; for $X=\left(x_{p,q}\right)$, $\mathrm{abs}(X)=(|x_{p,q}|)$. There is possibility that abs $\left\{{\mathrm{CSI}}^{\prime }\left(t_{j}\right)-{\mathrm{CSI}}^{\prime }\left(t_{i}\right)\right\}\approx 0$. In this case, CSI contains almost no information related to human activities even if user has made actions during this period of time.

To extract the components highly related to human activities, we find an optimum inner window $\left[t_{k},t_{l}\right]$ such that $t_{i}\leq t_{k}<t_{l}\leq t_{j}$ by maximizing a Frobenius norm of the CSI difference in each slide window:

(12) $$\begin{array}{c} t_{k},t_{l}=\underset{t_{k},t_{l}}{arg max}{∥{\mathrm{CSI}}^{\prime }\left(t_{l}\right)-{\mathrm{CSI}}^{\prime }\left(t_{k}\right)∥}_{F}. \end{array}$$

The Frobenius norm of the CSI difference indicates the intensity of human activities. The CSI difference $\Delta \mathrm{CSI}(t_{j})$ between time $t_{i}$ and $t_{j}$ is finally calculated over the optimum inner window:

(13) $$\Delta \mathrm{CSI}\left(t_{j}\right)=\mathrm{abs}\left\{{\mathrm{CSI}}^{\prime }\left(t_{l}\right)-{\mathrm{CSI}}^{\prime }\left(t_{k}\right)\right\}.$$

**Figure 6:**
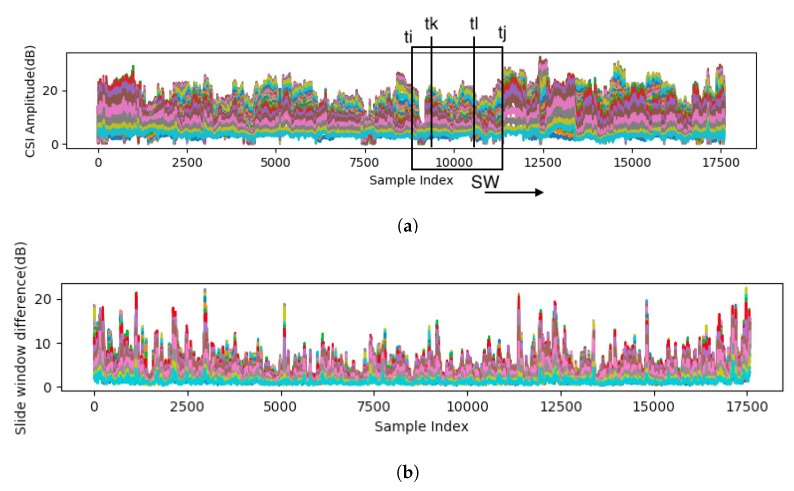
Example of slide windowing: (**a**) parameter and slide direction of slide window SW, and (**b**) calculated CSI difference $\Delta \mathrm{CSI}(t_{j})$ in each slide window.

Figure 6 shows an example of CSI difference $\Delta \mathrm{CSI}(t_{j})$ calculated in each slide window.

#### 3.4.2. Dominant Component Extraction

The dominant component extraction process extracts principal components related to human activities. The extraction is a two-step process. We first extract subcarriers sensitive to human activities and then remove small amplitude/phase changes.

As we can see in Figure 6, some subcarriers are almost constant. These subcarriers hardly change, regardless of whether human activity is intense or not because they are not sensitive to human activities. We exclude these insensitive subcarriers to improve classification accuracy and training speed.

The sensitive subcarriers are determined based on mean amplitude of the low-pass filtered calibrated channel response of each subcarrier. We first define channel response ${\tilde{H}}_{s}\left(t\right)$ of subcarrier *s*:

(14) $${\tilde{H}}_{s}\left(t\right)=\left[\begin{array}{cccc} {\tilde{H}}_{1,1,s}\left(t\right) & {\tilde{H}}_{1,2,s}\left(t\right) & \cdots & {\tilde{H}}_{1,m,s}\left(t\right)\\ {\tilde{H}}_{2,1,s}\left(t\right) & {\tilde{H}}_{2,2,s}\left(t\right) & \cdots & {\tilde{H}}_{2,m,s}\left(t\right)\\ \vdots & \vdots & \ddots & \vdots \\ {\tilde{H}}_{n,1,s}\left(t\right) & {\tilde{H}}_{n,2,s}\left(t\right) & \cdots & {\tilde{H}}_{n,m,s}\left(t\right) \end{array}\right],$$

where ${\tilde{H}}_{n,m,s}\left(t\right)$ is the low-pass filtered calibrated CSI of the *s*-th subcarrier between *n*-th transmission and *m*-th reception antennas at time *t*. A Frobenius norm $∥{\tilde{H}}_{s}\left(t\right)∥$ is then calculated and averaged over time for each subcarrier. We finally extract the *k*-largest subcarriers.

**Figure 7:**
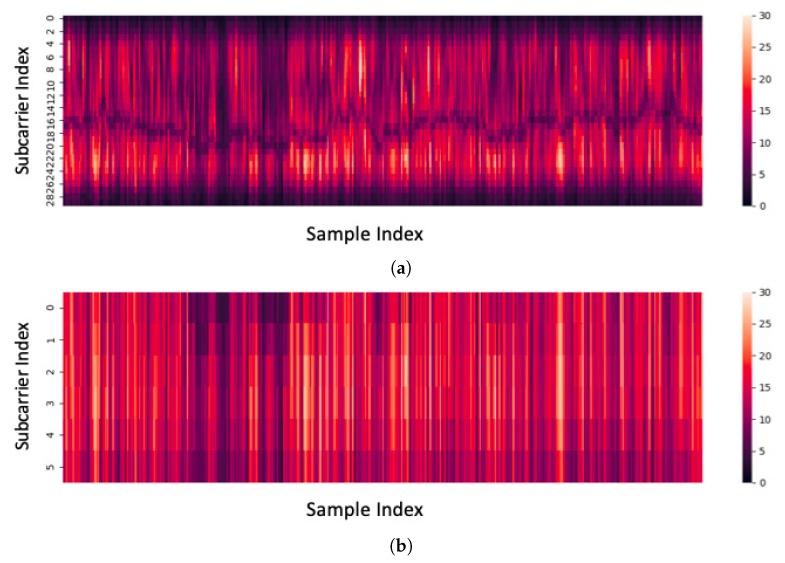
Sensitive subcarrier extraction: (**a**) amplitude of all subcarriers, and (**b**) amplitude of *k* most sensitive subcarriers (k=6).

Figure 7 shows an example of the sensitive subcarrier extraction. Figure 7a shows amplitude, i.e., Frobenius norm, of all subcarriers and Figure 7b shows the extracted subcarriers (k=6). As shown in the figure, the extracted subcarriers have relatively high amplitude values.

After the sensitive subcarrier extraction, we remove small changes in amplitude or phase to exclude outliers. Although we focus on the largest changes of CSI by slide windowing as described in Section 3.4.1, there are still outliers with significantly small changes compared to other windows. We remove these small changes by thresholding. We select first quartile $Q_{1}$ of the whole waveform of each element in the CSI difference matrix ΔCSI(t) as the threshold. The amplitude or phase of elements in ΔCSI(t) after peak extraction is:

(15) $$X_{i}\left(t\right)=\left\{\begin{array}{cc} \Delta X_{i}\left(t\right), & \Delta X_{i}\left(t\right)\geq Q_{1}\left\{\Delta X_{i}\left(t\right)\right\},\\ Q_{1}\left\{\Delta X_{i}\left(t\right)\right\}, & \Delta X_{i}\left(t\right)<Q_{1}\left\{\Delta X_{i}\left(t\right)\right\}, \end{array}\right.$$

where $X_{i}\left(t\right)$ is amplitude or phase of sensitive subcarriers in ΔCSI(t).

#### 3.4.3. Anomaly Detection

The anomaly-detection process in the danger detection block estimates whether a user is in *danger*. We also do not limit an anomaly-detection algorithm to use in the danger detection block. In this paper, one-class SVM is again employed. We train the one-class SVM classifier using the data obtained while a user is taking a shower and bathing as inlier data.

#### 3.4.4. Smoothing

We apply a smoothing filter to finally detect dangerous situations because the accuracy of anomaly detection is not 100%. We estimate user’s situation for each slide window and average the estimated results to finally determine the user’s situation. Our danger-pose detection system finalizes that the user is in a danger situation when the anomaly-detection processes estimates the user is in danger for specific duration of time in an observation window.

In a specific observation window, let S be a set of situations derived in each slide window from anomaly-detection processes in activity classification or danger detection blocks. x∈S can be not_in_bath, safe, or danger. Let $S_{\mathrm{not}\_\mathrm{in}\_\mathrm{bath}}$, $S_{\mathrm{safe}}$, and $S_{\mathrm{danger}}$ be subsets of the each estimated situation.

The smoothing process finalizes that the user is in danger when two equations below are both satisfied:

(16a) $$\begin{array}{cc} \frac{|S_{\mathrm{not}\_\mathrm{in}\_\mathrm{bath}|}}{\left|S\right|} & \leq t_{1}, \end{array}$$

(16b) $$\begin{array}{cc} \frac{|S_{\mathrm{danger}}|}{\left|S\right|} & >t_{2}, \end{array}$$

where $t_{1}$ and $t_{2}$ are two thresholds as we use the two-step estimation. The first equation finalizes that the user is surely in a bathtub, and the second equation finalizes that the user is in a danger situation. Threshold $t_{1}$ and $t_{2}$ are determined based on preliminary experiment results and are set to be $t_{1}=0.7$ and $t_{2}=0.2$ in our case.

## 4. Experiment

To evaluate the basic performance of the danger-pose detection system, we evaluated danger-pose detection performance using data collected in a bathroom of an apartment.

### 4.1. Experiment Setup

**Figure 8:**
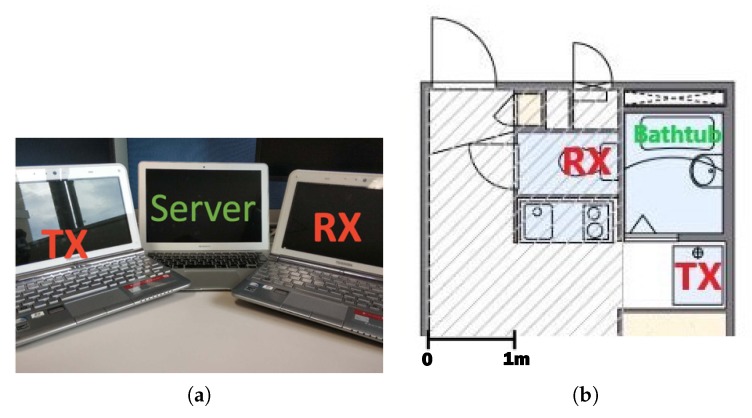
Experiment setup: (**a**) transmitter (TX), receiver (RX), and server used in our experiment, and (**b**) configuration of TX, RX, and bathtub.

Figure 8a shows equipment used in the experiment. Two Toshiba Dynabook laptops equipped with Intel 5300 NIC were used to transmit and receive Wi-Fi signals. Apple MacBook Air laptop equipped with 1.4 GHz Intel Core i5 processor and with 8 GB 1600 MHz DDR3 memory worked as a server to control transmitter and receiver, which was also used for danger-pose detection calculations.

Figure 8b shows experiment setup. We put transmitter (TX) and receiver (RX) outside of the bathroom. One subject human volunteered our experiment. We asked the subject to do three tasks: take a shower in a bathroom, take a bath normally, and simulate the danger poses in a bathtub. While the subject was doing each task, we controlled transmitter and receiver to collect CSI data. Although there were no other people in the experiment environment except the subject and an operator of the system, many people were living in other rooms in the apartment. The apartment also faces a road with normal traffic volume. The experiment was most likely affected by other people living in the apartment, passing vehicles, and pedestrians.

**Figure 9:**
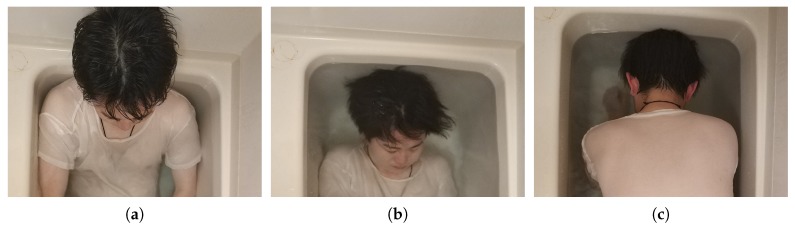
Simulated three dangerous situations: (**a**) steady lying position, (**b**) the whole body sinks below the water surface, and (**c**) the face sinks below the water surface.

For the dangerous situation simulation, we assumed three dangerous situations as shown in Figure 9: (1) keep the lying position in a long time, (2) sink the whole body below the water surface, and (3) sink the face below the water surface. The danger-pose detection system is used to detect dangerous situations when taking a bath. Although target dangerous situations are not limited to the three simulated situations, we conducted evaluations with these three situations to demonstrate the basic performance of our system as an initial evaluation.

We collected CSI data at a rate of 20 Hz on channel 40 in a 5-GHz band for couple of hours on six different days during a three-month period. During the three-month period, environmental changes including location of furniture and daily objects have been occurred. Locations of transmitter and receiver might also include errors up to approximately 10 centimeters as we put transmitter and receiver on each of the six days. For *not in bath*, *safe*, and *danger* activities, we collected 119,324, 288,291, and 21,572 CSI data samples, respectively, in total in six days.

Using the collected data, we performed six-fold leave-one-day-out cross validation. We trained one-class SVM classifiers in activity recognition and danger detection blocks using five-day data of the six-day data and evaluated classification performance with the remaining one-day data. We repeated this evaluation for six times to test all the data samples. The one-class SVM classifiers in the activity classification and danger detection blocks were trained with *not in bath* and *safe* data as inlier data, respectively. Please note that sensitive subcarriers in the dominant component extraction process were also determined with the five-day training data on each trial.

We evaluated classification performance by precision, recall, and F1-score, which are commonly used in detection performance evaluations, defined as:

(17) $$\begin{array}{cc} \mathrm{Precision} & =\frac{\mathrm{TP}}{\mathrm{TP}+\mathrm{FP}}, \end{array}$$

(18) $$\begin{array}{cc} \mathrm{Recall} & =\frac{\mathrm{TP}}{\mathrm{TP}+\mathrm{FN}}, \end{array}$$

(19) $$\begin{array}{cc} F1 & =\frac{2\cdot \mathrm{Precision}\cdot \mathrm{Recall}}{\mathrm{Precision}+\mathrm{Recall}}, \end{array}$$

where TP, FN, FP are the numbers of true positives, false negatives, and false positives, respectively. We especially focus on recall because high recall indicates small number of false negatives, i.e., small number of undetected danger situations.

### 4.2. Classification Performance of Each Block

We first evaluated classification performance of the activity classification and danger detection blocks for each slide window. *in bath* and *danger* are defined as positive detections in activity classification and danger detection blocks, respectively. For the danger detection block, we only feed *safe* and *danger* data in this evaluation as we evaluate the micro-benchmark.

Table 1 shows classification results of activity recognition and danger detection blocks. Please note that the result of the danger detection block is raw result without the smoothing process. From Table 1, we can confirm that the activity classification block exhibited high detection performance. Although the danger detection block showed relatively low detection performance in terms of F1-score, high recall indicates that the danger detection block successfully detected danger situations with small number of undetected danger situations.

We also measured computation time for danger-pose detection. The average computation time from pre-processing to activity classification was 12.96 milliseconds. Please note that the computation time for danger detection is evaluated in the following subsection as total computation time because the danger detection also relies on pre-processing block.

We next evaluated the influence of false positive detections in the activity recognition block, which are fed into the danger detection block. 5174 false positive detections in the activity recognition block are classified into *safe* or *danger*, both are incorrect classifications.

Table 2 shows the classification result of the danger detection block for false positive detections in the activity recognition block. We believe that the false positives in the activity classification block should be classified into *safe* in the danger detection block. On this assumption, precision, recall, and F1-score are changed to be 52.94%, 92.15%, and 67.25%, respectively. High precision of the activity classification block resulted in small influence on the performance of the danger detection block.

### 4.3. Total Danger Detection Performance

Finally, we evaluated the total danger detection performance, which is the danger detection result after smoothing over output of the danger detection block. We performed danger detection every 20 s using 400 samples of CSI data, i.e., an observation window size was 20 s. We divided collected data into observation windows for each activity. The number of the observation windows for *not in bath*, *safe*, and *danger* activities were 298, 720, and 53, respectively.

Table 3 shows danger-pose detection result including smoothing. Comparing Table 3 with the results of precision described in the last of Section 4.2, we can easily understand that the false positive detections were drastically reduced with the smoothing process while maintaining high recall. We can conclude that our danger-pose detection system successfully detected danger situations with a high recall of 96.23%.

Total computation time for danger-pose detection was also measured and averaged. The average total computation time was approximately 5.20 s for danger-pose detection on each observation window. Please note that we used MacBook Air for the detection, which has limited computational resources. The computation time can be easily reduced by using shorter observation window and by using high performance computer.

## 5. Conclusions

In this paper, we present a Wi-Fi CSI-based danger-pose detection system that can be used in a bathroom, especially in bathtub. Our danger-pose detection system performs two-step estimation: a user location is coarsely estimated with static features and then danger situations are detected with dynamic features. Because CSI data in danger situations is difficult to collect, we use an anomaly-detection algorithm that requires small amount or no data collected in danger situations for training. We conducted experimental evaluations with the data collected in an actual bathroom in an apartment with many residents on six separate days during a three-month period and demonstrated that our danger-pose detection system successfully detected danger situations with a recall of 96.23% and with the average computation time of 5.20 s for each observation window with a normal laptop. The computation time can easily be improved with a better performance computer with shorter observation window. We experimentally proved the feasibility of bathroom danger detection using wireless signals, but the amount of data for evaluation was limited. We plan to conduct more comprehensive experiments to surely validate the effectiveness of our system.

## Figures and Tables

**Table 1:** Classification results for (a) activity recognition and (b) danger detection blocks.

(a)				(b)			
	Estimated	*in Bath*	*not in Bath*		Estimated	*Danger*	*Safe*
Actual				Actual			
*in bath* (Positive)		294,402	15,461	*danger* (Positive)		19,878	1694
*not in bath* (Negative)		5174	114,150	*safe* (Negative)		14,874	273,417
Precision			98.27%	Precision			57.20%
Recall			95.01%	Recall			92.15%
F1			96.61%	F1			70.58%

**Table 2:** Classification result of danger detection block for 5174 false positive detections in activity classification block.

	Estimated	*Danger*	*Safe*
Actual			
*not in bath*		2377	2797

**Table 3:** Danger detection performance of the danger-pose detection system.

	Estimated	*Danger*	*Not Danger*
Actual			
*danger* (Positive)		51	2
not *danger* (Negative) (= *not in bath* or *safe*)		10	1008
Precision			83.61%
Recall			96.23%
F1			89.47%

## Abbreviations

The following abbreviations are used in this manuscript:

CSI	channel state information
FFT	fast Fourier transform
FN	false negative
FP	false positive
LOS	line-of-sight
MIMO	multiple input multiple output
NLOS	non-line-of-sight
OFDM	orthogonal frequency division multiplexing
PCA	principal components analysis
RSS	received signal strength
SVM	support vector machine
TP	true positive
